# Digital multiplexed analysis of circular RNAs in FFPE and fresh non‐small cell lung cancer specimens

**DOI:** 10.1002/1878-0261.13182

**Published:** 2022-02-10

**Authors:** Carlos Pedraz‐Valdunciel, Stavros Giannoukakos, Nicolas Potie, Ana Giménez‐Capitán, Chung‐Ying Huang, Michael Hackenberg, Alberto Fernandez‐Hilario, Jill Bracht, Martyna Filipska, Erika Aldeguer, Sonia Rodríguez, Trever G. Bivona, Sarah Warren, Cristina Aguado, Masaoki Ito, Andrés Aguilar‐Hernández, Miguel Angel Molina‐Vila, Rafael Rosell

**Affiliations:** ^1^ Germans Trias I Pujol Research Institute Badalona Spain; ^2^ 16719 Department of Biochemistry, Molecular Biology and Biomedicine Autonomous University of Barcelona Spain; ^3^ 16741 Department of Genetics University of Granada Spain; ^4^ 16741 Andalusian Research Institute in Data Science and Computational Intelligence University of Granada Spain; ^5^ Laboratory of Oncology Pangaea Oncology Barcelona Spain; ^6^ NanoString Technologies Seattle WA USA; ^7^ UCSF Helen Diller Family Comprehensive Cancer Center University of California San Francisco CA USA; ^8^ Department of Surgical Oncology Research Institute for Radiation Biology and Medicine Hiroshima University Japan; ^9^ Oncology Institute Dr. Rosell IOR Quirón‐Dexeus University Institute Barcelona Spain; ^10^ 16719 Autonomous University of Barcelona Spain

**Keywords:** biomarkers, cancer, circRNA, diagnosis, nCounter, NSCLC

## Abstract

Although many studies highlight the implication of circular RNAs (circRNAs) in carcinogenesis and tumor progression, their potential as cancer biomarkers has not yet been fully explored in the clinic due to the limitations of current quantification methods. Here, we report the use of the nCounter platform as a valid technology for the analysis of circRNA expression patterns in non‐small cell lung cancer (NSCLC) specimens. Under this context, our custom‐made circRNA panel was able to detect circRNA expression both in NSCLC cells and formalin‐fixed paraffin‐embedded (FFPE) tissues. CircFUT8 was overexpressed in NSCLC, contrasting with circEPB41L2, circBNC2, and circSOX13 downregulation even at the early stages of the disease. Machine learning (ML) approaches from different paradigms allowed discrimination of NSCLC from nontumor controls (NTCs) with an 8‐circRNA signature. An additional 4‐circRNA signature was able to classify early‐stage NSCLC samples from NTC, reaching a maximum area under the ROC curve (AUC) of 0.981. Our results not only present two circRNA signatures with diagnosis potential but also introduce nCounter processing following ML as a feasible protocol for the study and development of circRNA signatures for NSCLC.

## Graphical abstract

Aberrant circular RNA (circRNA) expression is present in lung cancer. Using nCounter with machine learning, we discovered two signatures able to discriminate FFPE lung cancer samples from controls even at early stage. Our results not only highlight the potential of circRNAs as lung cancer biomarkers but also introduce nCounter as a suitable platform for circRNA expression studies in these samples.

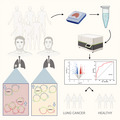

**Abbreviations**
AUC: area under the curve
circRNA: circular RNA
FFPE: formalin‐fixed paraffin‐embedded
GBM: gradient boosting machines
KNN: k‐nearest neighbors
LOOCV: leave‐one‐out cross‐validation
miRNA: micro RNA
ML: machine learning
NPV: negative predictive value
NSCLC: non‐small cell lung cancer
PCR: polymerase chain reaction
PPV: positive predictive value
RF: random Forest
RFE: recursive feature elimination
RNAseq: RNA sequencing
ROC: receiver operating characteristic
RT‐qPCR: quantitative reverse transcription PCR
SD: standard deviation

## 1 Introduction

circRNAs are a newly re‐defined type of endogenous RNA molecules originated by a noncanonical process called ‘back‐splicing’. Through this mechanism, the 5′ splice donor covalently links to the 3′ end of an upstream exon, resulting in a single‐stranded circular structure which can include one or different exonic/intronic regions [1]. This particular assembly lacking a poly(A) tail makes them very stable and resistant to exonuclease‐mediated degradation when compared to their linear counterparts [2].

The existence of circRNAs has been acknowledged for more than 45 years. First evidence was reported in 1976 with the first description of viroids as ‘single‐stranded and covalently closed circular RNA molecules’ [3], and their discovery in humans followed almost two decades later [4]. However, it is not until recently that their role has been clarified, evolving from abnormally spliced unfunctional ‘scrambled’ transcripts to circular RNA molecules with a marked role in homeostasis [5, 6].

CircRNAs have been classified as noncoding RNA for many years, due to the lack of a 5′ cap structure and their inability to bind to ribosomes. However, recent studies reported that some circRNAs can be translated into small functional peptides in a cap‐independent manner [7]. Other functions may include serving as protein decoys, scaffolds, and/or recruiters [8], or regulating the canonical transcription by competing with the formation of linear cognates via back‐splicing [9, 10]. Nonetheless, the most well‐studied function is their interaction with miRNAs. A single circRNA can have several miRNA‐binding sites through which targeted miRNAs get ‘sponged’, thereby blocking their activity [11]. It is throughout this mechanism how they predominantly exert their role as cell proliferation regulators targeting mediators of classical signaling pathways, such as MAPK/ERK, PI3K/AKT, and WNT/β‐catenin, or cell cycle checkpoint regulators [11]. Because of their implication in the above‐mentioned processes, dysregulation of circRNA expression can be associated to the development of different malignancies, including lung cancer. CircRNAs are significantly associated with tumorigenesis, proliferation, migration, and sensitivity to lung cancer therapies [12] and, as a result, have been presented in many recent studies as novel biomarkers to assess disease status.

However, the number of studies focusing on the development of circRNA signatures with either diagnostic or prognostic value in human malignancies is rather small, probably due to the lack of standardized circRNA quantification methods, which in turn is hampering the development of clinically applicable assays. RT‐qPCR is widely used as a quantification tool for circRNA expression studies. While its sensitivity and short turnaround time proves beneficial for circRNA research, several events such as template switching, rolling circle amplification, or the bias attached to this technique may hinder the results [13]. In addition, it does not allow high‐throughput analysis, which is necessary for biomarker discovery. Microarrays or RNAseq may overcome these limitations; however, the first have a limited range of detection disregarding those targets with either very low or high expression, while the latter not only results rather expensive but also includes other restrictions such as the use of long time‐consuming protocols, or complex data analysis [14, 15].

The nCounter technology allows multiplex analysis of up to 800 transcripts by direct capture and counting of individual targets [16]. With a short turnaround time and minimal hands‐on work, it provides results in < 48 h with the use of an intelligible software. However, despite the growing number of laboratories using this platform, it still gets mostly restricted to mRNA analysis.

In this proof‐of‐concept study, we retrospectively analyzed the circRNA expression profiles in NSCLC cell lines and FFPE tissues by using a custom‐designed 78 circRNA nCounter panel. Our data demonstrate that nCounter can be employed not only for basic circRNA research but also for the development of clinically useful circRNA signatures.

## 2 Materials and methods

### 2.1 Patient samples and cell lines

This study was carried out in accordance with the principles of the Declaration of Helsinki, under an approved protocol of the institutional review boards of Quirón Hospitals, and the IGTP‐HUGTP Biobank. FFPE lung cancer tissues were retrospectively collected from 27 early‐stage and 26 late‐stage cancer patients from the different Quirón hospitals (Table 1). FFPE tissue samples from 16 donors were collected as controls from the IGTP‐HUGTP Biobank. Most controls did not present any type of cancer, except for four samples which were extracted from the nontumorigenic region of the lung from a cancer patient. Individuals with different pathologies were also included to ensure the development of signatures specific of lung cancer (Table S1).

**Table 1:** Clinicopathologic characteristics of enrolled patients (*n* = 69). NSCLC, non‐small cell lung cancer.

Clinicopathological characteristics	Lung cancer patients (*n* = 53)	Noncancer controls (*n* = 16)
Gender—no. (%)		
Male	28 (52.8)	10 (62.5)
Female	25 (47.2)	6 (37.5)
Age—years		
Median	66	59
Range	32–85	29–76
Smoking status—no. (%)		
Ex‐ or current smoker	40 (75.5)	9 (56.25)
Never smoker	11 (20.8)	5 (31.25)
Not information	2 (3.7)	2 (12.5)
Histological type		
Adenocarcinoma	43	–
Squamous carcinoma	1	–
Other NSCLC	9	–
Driver mutation		
EGFR	6	–
Exon19	3	–
Exon21	1	–
Exon20‐21	1	–
Exon21 and amplification	1	–
KRAS	12	–
G12A	2	–
G12C	3	–
G12V	4	–
G12R	1	–
Other	2	–
BRAF	1	–
ROS	1	–
RET	2	–
ALK	1	–
MET (exon14 mutation)	1	–
Other alterations	5	
Not information	24	–
Tumor stage—no. (%)		
I	16 (30.2)	–
II	4 (7.5)	–
IIIA	7 (13.2)	–
IIIB	3 (5.6)	–
IV	23 (43.4)	–

All collected samples were assessed for tumor and lymphocyte infiltration by a pathologist (Table S2).

Written informed consent was obtained from all patients and further documented; samples were de‐identified for patient confidentiality. Clinical information collected from each patient was limited to gender, age, smoking status, tumor histology, driver mutation, and stage.

A panel of seven human lung cancer cell lines harboring different mutations was selected along with two normal epithelial cell lines (Table 2). Cell lines were maintained following standard culture conditions [17] in RPMI‐1640 or DMEM (Gibco, Life Technologies, Carlsbad, CA, USA) supplemented with 10% fetal bovine serum (Gibco). All cell lines were tested for mycoplasma infection.

**Table 2:** Characteristics of the lung cell lines included in the study. AD, adenocarcinoma; ATCC, American Type Culture Collection; NE, normal epithelial; UCSF, University California San Francisco; UTSW, University of Texas Southwestern.

Cell line	Histology	Gene	Mutation	Origin
A549	AD	*KRAS*	G12S	ATCC
HOP‐62			G12C	ATCC
PC9		*EGFR*	E746_A750 DL	Hoffmann‐La Roche, with the authorization of Dr. Mayumi Ono
HCC‐827			E746_A750 DL	ATCC
NCI‐H1666		*BRAF*	G466V	ATCC
NCI‐H2228		*ALK*	*EML4‐ALK*, variant 1	ATCC
NCI‐H3122			*EML4‐ALK*, variant 3	ATCC
AALE	NE	–	wt	Dr. Trever Bivona Lab, UCSF
HBEC30KT				Dr. Minna Lab, UTSW

### 2.2 RNA extraction

RNA extraction was performed following previously published methods [18, 19]. RNA from fresh cell lines was isolated using the Allprep DNA/RNA/miRNA universal kit (Qiagen, Hilden, Germany). FFPE cells and tissues were deparaffined with xylene. After the removal of xylene using ethanol, RNA was extracted using the High Pure FFPET RNA isolation Kit (Roche, Rotkreuz, Switzerland). RNA quantification was performed using the Qubit 4 Fluorometer (Invitrogen, Carlsbad, CA, USA) with the Qubit RNA HS Assay Kit (Invitrogen). RNA integrity was assessed with the 2100 Bioanalyzer system (Agilent Technologies, Santa Clara, CA, USA) using the RNA 6000 Nano kit (Agilent Technologies).

### 2.3 Rnase‐R treatment

5 µg of total RNA was either treated or mock‐treated with RNase‐R (Lucigen, Madison, WI, USA). RNA samples were denatured at 95 °C for 30 s following addition of a master mix containing RNase‐R (or molecular grade water in the case of mock‐treated samples), 10× RNase‐R buffer adjusted to the final volume, and molecular grade water. Samples were incubated 160 min at 40 °C and kept at 4 °C prior RNA quantification and subsequent nCounter hybridization.

### 2.4 RT‐qPCR and Sanger sequencing analysis

RT‐qPCR and Sanger sequencing of circRNA junction sites were performed as previously described [18]. 10 µL of total RNA was converted into cDNA using the M‐MLV reverse transcriptase enzyme and random hexamers (Invitrogen).

A 1 : 3 dilution of cDNA was performed, and 2.5 µL were added to the Taqman Universal Master Mix (Applied Biosystems) in a 12.5 µL reaction containing a specific pair of primers and probe for each gene. Three replicas of each sample were run for the quantification of the expression of each assessed circRNA. Three replicas of each sample were run for the quantification of the expression of each assessed circRNA. Divergent primers and probe sets were designed using Primer Express 3.0 Software (version 3.0.1, Applied Biosystems) with the latter spanning the circRNA junction site (Table 3). Quantification of gene expression was performed using the QuantStudioTM 6 Flex System (Applied Biosystems) and calculated according to the comparative Ct method.

**Table 3:** Primer and probe design for circRNA validation by RT‐qPCR. In blue marked the junction site.

circRNA		
circEPB41L2 (hsa_circRNA_0001640)	Forward	GAAGACCAAAACTGTCCAGTGTAAAG
	Reverse	CACTTCAGACACAGAGCCTACTTCA
	Probe	TGACCTGGAGCATAAG
circSOX13 (hsa_circRNA_0004777)	Forward	CAGTGACTGGAAGGAGAGGTTTC
	Reverse	CTGGGCAGAGATGGGGCT
	Probe	AAAGATGTCAAAGGATGTCCATGA
circBNC2 (hsa_circ_0086414)	Forward	GTCTGCACAGTGGCTGGTTG
	Reverse	GGTGATGATTTCCTCTTCTCGAG
	Probe	AGACAGGATGCTGCTG

In all quantitative experiments, a sample was considered not evaluable when the standard deviation of the *Cq* values was > 0.30 in two of the three independent analyses (*n* = 3).

For Sanger sequencing, 10 µL of each PCR product was loaded on a Precast Agarose HT‐1gel and visualized under UV light (E‐Gel™ Safe Imager™ Real‐Time Transilluminator, Invitrogen) after electrophoresis (E‐Gel™ iBase™ Power System, Invitrogen).

Five microliters of each cDNA sample were purified using the PCR ExoSAP‐IT Product Clean up Reagent (Applied Biosystems). Sequencing PCRs were set up using the BigDye Terminator v3.1 Cycle Sequencing Kit (Applied Biosystems), forward primer, cDNA and water in a final volume of 20 µL. Sequencing PCR was performed using a Verity 96 well thermal cycler (Applied Biosystems).

After sequencing amplification, samples were loaded into a 96‐well plate and subjected to Sanger sequencing using the 3130 Genetic Analyzer (Applied Biosystems).

### 2.5 miRNA prediction and circRNA‐miRNA network construction

MiRNAs targeted by the differentially expressed circRNAs found in early‐stage FFPE lung cancer tissues were predicted using the circinteractome tool (https://circinteractome.nia.nih.gov). circRNA‐miRNA interaction network was built using cytoscape (v3.8.2; https://cytoscape.org). Association of miRNAs with cancer‐associated downstream signaling pathways was investigated using the miRCancer database (https://mircancer.ecu.edu).

### 2.6 NanoString nCounter panel design and sample processing

A custom‐made panel of 78 circRNAs was produced, including both highly and lowly expressed circRNAs that could be related to lung cancer (Table S3). Each probe was designed to target a flanking exonic sequence between 35–55 nucleotides of the circRNA junction site. They also contain a complementary region to capture and reporter probes, conforming a precise configuration that allows specific recognition of circular transcripts (Fig. S1). In addition, six linear reference genes (GAPDH, MRPL19, PSMC4, RPLP0, SF3, and UBB) and four mRNAs of FAM13B, HIPK3, MGA, and UBXN7 genes were included (Table S2).

Sample processing in the nCounter was performed as previously described [18] following NanoString's guidelines (Fig. S2).

### 2.7 Data normalization and differential expression analysis

Raw count values were exported to Microsoft Excel (version 16.40, Microsoft, Redmond, WA, USA) using nSolver Analysis Software (version 4.0.70, NanoString Technologies, Seattle, WA, USA). For each of the circRNAs included in the panel, raw counts lower than the cut‐off value established as background were automatically excluded from further analysis. Background was calculated for each sample by using the mean of the negative probe counts plus two times the standard deviation. Only circRNAs with a value > 10 counts after background subtraction were considered as expressed. Subsequent circRNA‐specific counts were normalized by dividing this number by the total number of counts for this sample. Resulting number was multiplied by 10 000 (units expressed in counts per 10 000).

Further differential expression analysis of raw nCounter data was carried out with r (version 4.0.2; R Core Team and the R Foundation for Statistical Computing, Vienna, Austria) and R studio (version 1.3.1056; RStudio PBC, Boston, MA, USA). Technical variability correction, normalization, and differential expression analysis was performed using the ruvseq (version e1.24.0; Bioconductor Core Team, Buffalo, NY, USA) and deseq2 (version 1.30.0; Bioconductor Core Team) packages (ruvseq‐deseq2, Bioconductor Core Team). Firstly, the RUVg function was used to estimate the unwanted variation among samples based on the positive controls. The positive controls used in the NanoString panel are Spike‐In control sequences; therefore, analogous constant expression of positive controls is expected across all samples. Secondly. DESeq2 was used to perform the normalization of the data, while accommodating the estimated factors provided by the RUVg function. Finally, DESeq2 was used to perform hypothesis testing in order to identify differentially expressed circRNAs. Shrunken log_2_ fold‐change (log_2_FC) was then reported by DESeq2 along with adjusted *P*‐values. Batch effect was considered during normalization using RUVSeq‐DESeq2. The normalized data were employed for ML techniques.

Volcano plots were used to visualize log_2_FC on the *x*‐axis and −log_10_ adjusted *P*‐values on the *y*‐axis.

### 2.8 Machine learning classification

Recursive feature elimination (RFE) was used to perform feature selection and the LOOCV algorithm was applied on the full panel of circRNA transcripts. The number of features to select was set by default at 4, 8, 16, and 78. The number of features that yielded best performance after cross‐validation was automatically selected. To test whether generated data had enough discriminative information to build a robust model for the classification of cancer samples from controls, different paradigms of classification models were tested to provide the most accurate results. Under this context, three classification approaches were performed with the selected features: an ‘instance‐based’ model (KNN). This model uses the distances among samples to obtain a predictive label; and two different ensemble mechanisms with decision trees—bagging (RF) and boosting (GBM).

For the analysis of early‐stage lung cancer samples versus control samples, GBM was excluded due to the high volume of samples is required for this model.

The model with the highest ROC AUC value was then selected as the final model. A confidence threshold of 0.5 was considered for the calculation of PPV and NPV. Additional statistical indicators such as accuracy, sensitivity, and specificity were also calculated.

## 3 Results

### 3.1 nCounter for circRNA detection in fresh NSCLC samples

Based on a literature review, 78 circRNAs were selected according to their differential expression in lung cancer specimens for the development of an nCounter panel (Table S3). To test the reproducibility of this panel for circRNA detection, RNA from fresh PC9 cells was subjected to nCounter analysis in three independent reactions. As a result, a strong correlation was found between the normalized counts for each individual circRNA, represented by a Spearman's *r* of 0.82–0.88, *P* < 0.01 (Fig. S3).

**Fig. 1:**
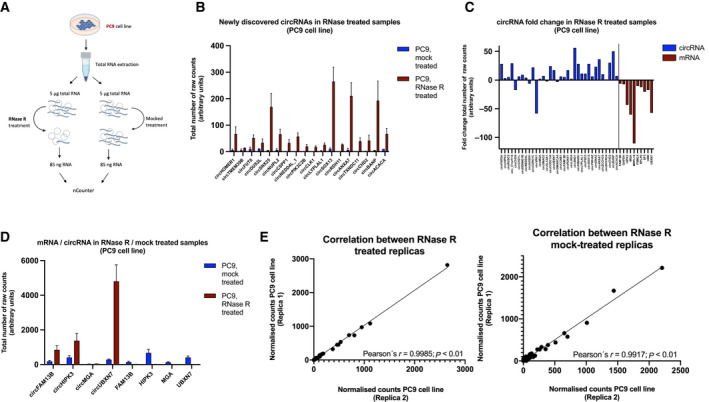
Analysis of circRNA from RNase‐R‐treated samples. (A) Workflow for circRNA enrichment with RNase‐R. (B) Representation of the newly discovered circRNAs after RNase‐R treatment. Bars indicate the mean of the replicas (*n* = 2). Error bars indicate SD. (C) CircRNA/linear HK fold‐change after RNase‐R treatment (*n* = 2). (D) Comparison of circRNAs/mRNA cognates in RNase‐R/mock‐treated samples. Bars indicate the mean of the replicas (*n* = 2). Error bars indicates SD. (E) Correlation of the nCounter replicas (*n* = 2) for each treatment. Pearson's coefficient is indicated.

Then, RNA from the same cell line was used in an experiment with RNAase‐R, an enzyme that degrades linear RNA, to elucidate if the nCounter probes bind specifically to the circRNA of the genes included in the panel (Fig. 1A). As a result, 18 new transcripts that could not be detected in mock‐treated samples were observed after RNase‐R treatment (Fig. 1B). In addition, among the 34 transcripts identified in both types of samples, the counts of 28 (82.3%) increased at least 2‐fold after RNAse‐R treatment. CircSND1 and circBANP were found with the highest enrichment, with a 56‐ and 33‐fold change, respectively. CircCHD9, circAASDH, circVRK1, circSLC8A1, and circSMARCA5 were the only circular transcripts affected by the exonuclease activity of RNase‐R, showing a lower number of counts after incubation with the enzyme (Fig. 1C). All mRNA controls, including the linear forms of FAM13B, HIPK3, MGA, and UBXN7, were found with reduced or null expression after treatment (Fig. 1D).

A high correlation was found between the two replicas included for each of the conditions (Pearson's *r* = 0.99917; *P* < 0.01 and *r* = 0.9985; *P* < 0.01 for mock‐treated and RNase‐R‐treated samples, respectively) demonstrating the specificity of the assay (Fig. 1E).

### 3.2 nCounter for circRNA detection in FFPE NSCLC samples

To assess the performance of our panel in FFPE samples, RNA from paired FFPE and fresh PC9 cell line was extracted and processed in the nCounter. The number of total raw counts in PC9 FFPE samples was significantly lower compared to fresh PC9 samples (771.870 versus 1.353.811). However, despite the suboptimal quality observed in the RNA extracted from the FFPE cells (Fig. S4), a statistically significant correlation was found when comparing both types of input (Fig. S5A).

Next, we assessed the feasibility of RNase‐R treatment in FFPE samples. As a result, overall circRNA enrichment was not achieved, in contrast to what was previously observed in RNA extracted from fresh cells. Most circRNAs were found to be degraded to different extents in RNase‐R‐treated replicas when compared with the controls, indicating that such treatment should be avoided when working with FFPE samples (Fig. S5B).

Then, different concentrations of FFPE‐derived RNA (between 250 and 2000 ng of total RNA) were tested assessing the effect on downstream nCounter analysis. As a result, saturation was not achieved with the highest concentration, suggesting that a greater RNA input could be applied. Analysis of normalized counts across all samples indicated similar performance of 250 ng compared to the rest of tested concentrations, with a Pearson's correlation between 0.99–1.00 (Fig. S6). As a result, 250 ng of total RNA was selected for the rest of the study.

### 3.3 circRNA expression in NSCLC fresh cell lines

A set of seven lung cancer cell lines were selected according to their driver mutation, along with two normal epithelial cell lines (Table 2). Duplicates of equal RNA concentrations were run in all cases.

**Fig. 2:**
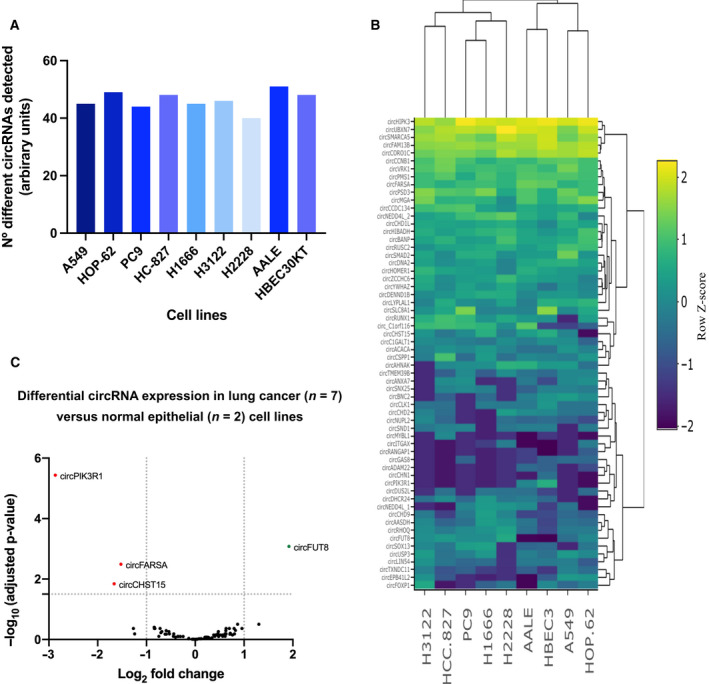
CircRNA analysis in lung cancer (A549, HOP‐62, PC9, HCC‐827, H1666, H3122, and H2228) and epithelial cells (AALE and HBEC30KT). (A) Bar plot representing total circRNAs detected in each of the cell lines. (B) Hierarchical clustering of cell lines based on circRNA expression. (C) Differential circRNA expression analysis of log_2_‐normalized counts between lung cancer and normal lung cells.

Out of the 78 circRNAs included in the panel, 33 were expressed in all cell lines. Nineteen were expressed in epithelial cells and not in all lung cancer cells, while only one, circFUT8 was only expressed in all lung cancer cell lines (Fig. S7). Nineteen circRNAs included in the panel were not found in any of the assessed cell lines. Fifty‐one was the highest number of circular transcripts displayed by any cell line (AALE). The NCI‐H2228 cell line showed the lowest number, with only 40 circRNAs detected (Fig. 2A). Overall, total raw counts were significantly higher in normal epithelial lung cell lines compared to cancer cell lines (Fig. S8). Hierarchical clustering led to a separation of the KRAS cell lines and normal epithelial cell lines from the rest (Fig. 2B). The two EGFR mutant cell lines positioned together, showing a distinctive group of downregulated circRNAs (circBNC2, circCLK1, circCHD2, and circNUPL2) compared to the rest of the cell lines.

Finally, differential expression analysis revealed four circRNAs that allowed for differentiation between the seven‐lung cancer cells and normal epithelial cells. CircPIK3R1, circFARSA, and circCHST15 were found downregulated in the cancer cell lines, while circFUT8 was upregulated (Fig. 2C).

### 3.4 circRNA expression in FFPE NSCLC versus nontumor tissue

**Fig. 3:**
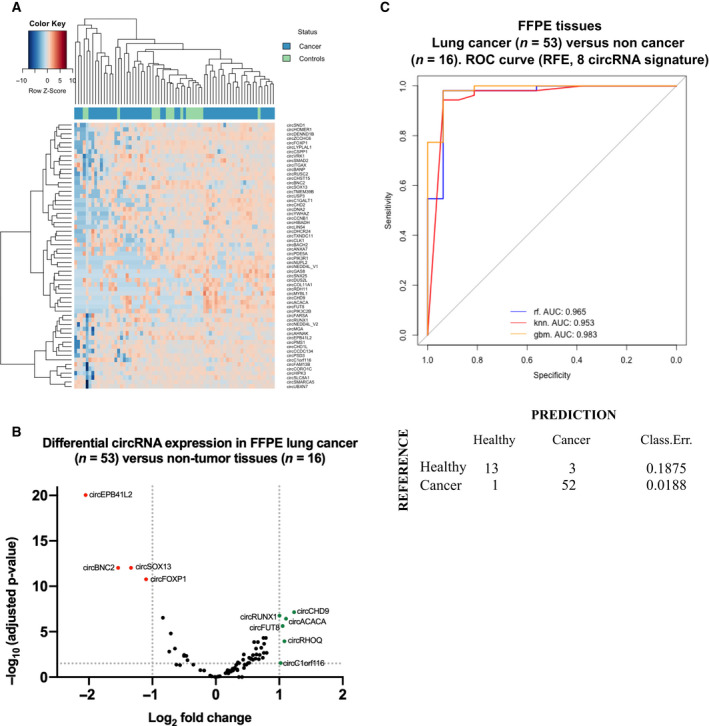
(A) Heatmap showing the circRNA expression in lung cancer and control specimens. Unsupervised clustering was performed based on total circRNA expression. (B) Volcano plot showing the circRNA log_2_ fold‐change in FFPE lung cancer (*n* = 53) versus control (*n* = 16) FFPE tissues. (C) Area under the ROC curve for the classification of lung cancer and control samples. Confusion matrix was generated based on the RF classification scores. Classification error scores are indicated.

A total of 53‐lung cancer samples and 16 control tissue samples were selected and processed with the circRNA nCounter panel. Initial analysis included normalization of counts for each circRNA as described in the methods section, followed by unsupervised hierarchical clustering of patient samples based on total circRNA expression. A partial separation between cohorts was achieved, indicating a group of circRNAs with discriminatory potential (Fig. 3A).

A differential expression analysis revealed a cluster of 10 differentially expressed circRNAs. CircEPB41L2, circBNC2, circSOX13, and circFOXP1 were downregulated in lung cancer tissues, while circRUNX1, circCHD9, circACACA, circFUT8, circRHOQ, and circC1ORF116 were overexpressed (Fig. 3B).

Additionally, we also investigated the possible differences in circRNA expression based on the smoking habits of the lung cancer cohort. As a result, four circRNAs (circCSPP1, circNEDD4L, circSOX13, and circCORO1C) negatively correlated with smoking status with *P* = 0.015, *P* = 0.043, *P* = 0.017, and *P* = 0.045 respectively (Student's *t*‐test).

Next, a ML approach was used to develop a circRNA signature predictive of lung cancer.

Due to the low number of samples to be analyzed (*n* = 59), we decided to use LOOCV as a validation model, which considers only one sample for testing in each interaction reducing the bias to the minimum when compared to other techniques such as stratified cross validation. As a result, a RFE algorithm selected an 8‐circRNA signature (including circSOX13, circEPB41L2, circFOXP1, circBNC2, circCORO1C, circCHD9, circSNX25, and circPIK3R1) as the final model, providing a ROC AUC of 0.965, 0.953, 0.983 with RF, KNN, and GBM classifiers, respectively (Fig. 3C). A PPV of 98.1% and NPV of 81.2% were achieved with the final model. The accuracy, sensitivity, and specificity of the signature were of 97.1%, 94.5%, and 92.8%, respectively.

#### 3.4.1 CircRNA expression in early‐stage NSCLC tissues

Next, the 27 early‐stage NSCLC samples (stages I–IIIA) of our cohort were compared to the 26 late‐stage specimens (stages IIIB and IV) (Table 1) to assess those differentially expressed circRNAs emerging early in the disease.

**Fig. 4:**
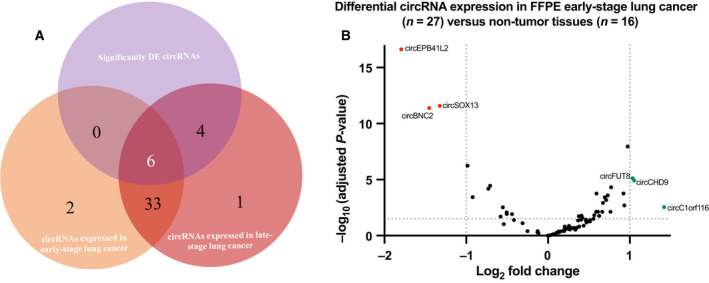
CircRNA expression in early‐stage NSCLC samples. (A) Venn diagram displaying circRNAs identified in early‐ and late‐stage samples, featuring those shared by both cohorts. DE circRNAs are indicated. (B) Differential expression analysis of log2‐normalized counts between the early‐stage lung cancer cohort (*n* = 27) and control (*n* = 16) FFPE tissues. circEPB41L2, circSOX13, and circBNC2 were found downregulated and circFUT8, circCHD9, and circ_C1orf116 were found upregulated as previously described with all stages of lung cancer.

**Fig. 5:**
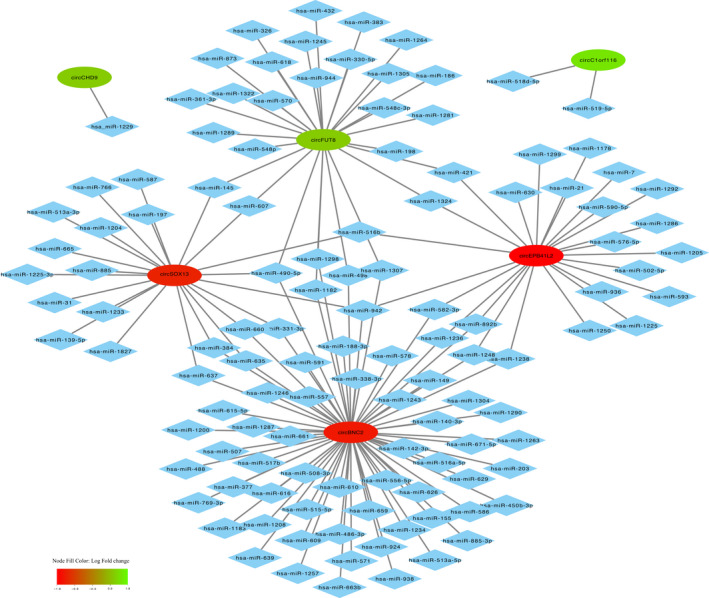
Mapping network showing predicted sequence‐pairing circRNA‐miRNA interaction of differentially expressed circRNA found in early‐stage lung cancer tissues. CircRNAs are represented by elliptic nodes and colored based on their log fold‐change. Complementary binding miRNAs are represented by diamond shaped nodes.

From the 41 circRNAs expressed in early‐stage samples, 39 were shared with late‐stage samples (Fig. 4A). Only 6 out of these 39 transcripts were differentially expressed when compared with the control specimens (Fig. 4B). Interestingly, one of these circRNAs (circFUT8) was found upregulated in both lung cancer tissues and lung cancer cell lines. To shed some light on the potential targets of these six circRNAs, a circRNA‐miRNA network was built based on sequence‐pairing prediction (Fig. 5). Using circinteractome database, 64 miRNAs were found to potentially bind to differentially expressed circRNAs, with 29 of them showing more than 1 binding site (Fig. S9).

**Fig. 6:**
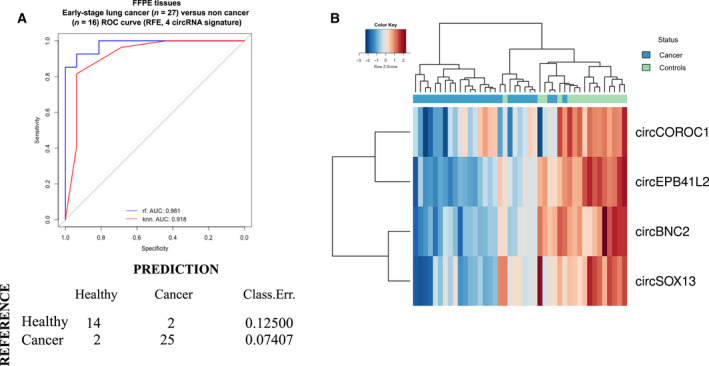
(A) Area under the ROC curve of the 4 circRNA‐signature using recursive feature elimination (RFE) for cohort classification. Confusion matrix was generated based on the RF classification scores. Classification error scores are indicated. (B) Hierarchical clustering of samples based on the 4‐circRNA signature.

Additional ML analysis was performed in early‐stage lung cancer and control samples. RFE algorithm provided a signature that included four circRNAs (circEPB41L2, circSOX13, circBNC2, circCORO1C) and provided a ROC AUC of 0.981, 0.918 with RF and KNN, respectively (Fig. 6A). PPV and NPV were of 92.6% and 87.5%, whereas accuracy, sensitivity, and specificity were of 90.6%, 92.6%, and 87.5%, respectively with the selected model. Hierarchical clustering based on the four circRNA included in the signature allowed a clear differentiation between both cohorts (Fig. 6B).

### 3.5 Univariate analysis related to lung cancer risk

**Fig. 7:**
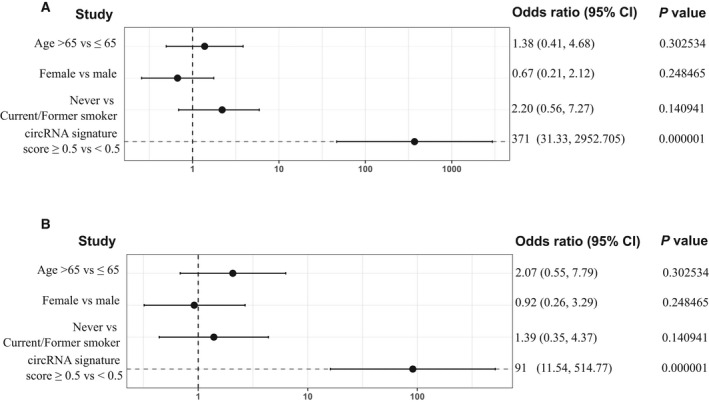
Univariate analysis exploring associations between patient characteristics and lung cancer to determine risk factor. Forest plot represents the odds ratios in (A) lung cancer; and (B), early‐stage lung cancer cohorts with a 95% Wald confidence limit. Student's t‐test was used for the calculation of *P*‐values.

We then explored if certain patient characteristics could provide risk factors for lung cancer by performing a univariate analysis (Fig. 7). Several characteristics that could be associated with higher risk of lung cancer such as age, gender, and smoking status were evaluated. No significant association could be found between lung cancer and any of the characteristics previously mentioned. However, presented signatures for lung cancer and early‐lung cancer classification were found to be significant predictive factors for lung cancer, with an odds ratio of 371 and 91, respectively.

### 3.6 Validation by RT‐qPCR and Sanger sequencing of circRNA junction sites

CircEPB41L2, circSOX13 and circBNC2 not only were significantly downregulated both in early and late stages showing the highest fold‐change, but also were selected by the RFE algorithm as part of the two predictive signatures. As a result, these 3 targets were selected to validate the nCounter performance using RT‐qPCR.

**Fig. 8:**
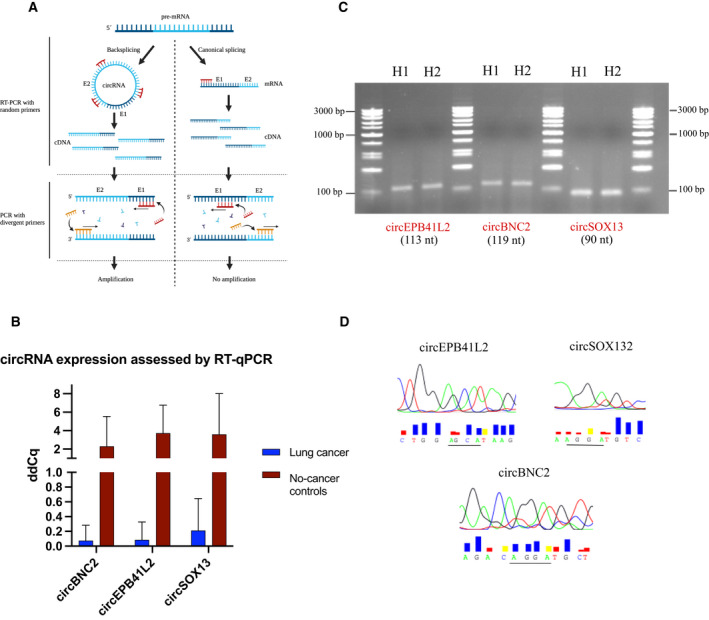
Validation of nCounter results by RT‐qPCR and further Sanger sequencing. (A) Representation of circRNA amplification using divergent primers. (B) Bar plot of RT‐qPCR results depicting downregulation of circEPB41L2, circSOX13, and circBNC2 in lung cancer versus control tissues validating previous nCounter results. Bars indicate the mean of the 10‐lung cancer (*n* = 3) and 10 control samples (*n* = 3). Error bars indicate SD. (C) Electrophoresis gel of amplified circEPB41L2 (113 nt), circBNC2 (119 nt) and circSOX13 (90 nt). (D) Sanger sequencing results spanning the junction site (underlined) of cited circRNAs.

Divergent primers and probes spanning the junction sites were designed for the specific amplification of cited circular transcripts (Fig. 8A) in 10 NSCLC and 10 control FPPE tissue samples previously assessed with the nCounter circRNA panel.

RT‐qPCR results correlated with the data previously obtained from nCounter, indicating downregulation of cited circRNAs in NSCLC samples (Fig. 8B). A gel electrophoresis of the PCR products revealed three bands corresponding to the size of expected amplicons (Fig. 8C). Further Sanger sequencing validated these findings by exposing the circRNA junction sites (Fig. 8D).

## 4 Discussion

Precision oncology currently relies on genomic, transcriptomic, or proteomic‐based features that serve as decision‐making support, predicting treatment outcome [20].

The re‐discovered role of circRNAs as regulatory entities of miRNAs, affecting the occurrence and development of different malignancies, has been supported by the growing number of studies that highlight their potential as cancer biomarkers and therapeutic targets in future personalized medicine [21]. Investigation of novel signatures based on these biomolecules could therefore be of interest to achieve earlier diagnosis by developing new tests or complimenting existing ones. However, the lack of standardized methods for their study is preventing their clinical validation and further implementation in the clinical practice.

The nCounter platform allows multiplexed digital gene expression analysis by direct counting of RNA molecules. With a wide use for transcriptomic studies, nCounter has been recently adapted for the detection of circRNAs using a specific probe design where sequences span the circRNAs junction site [19]. On this regard, some authors have proved the benefits of this technology to study circRNA subcellular distribution [22], or elucidate the potential roles in skin [23, 24] or brain diseases [25]. However, to date, no one has explored this platform in FFPE samples for the development of lung cancer signatures.

Here, we prove the use of nCounter for circRNA studies in FFPE lung cancer tissues and cell lines, developing a protocol for their study.

Due to the lack of any commercially available nCounter circRNA panel, we first performed an extensive literature research, looking for circular RNA candidates described to be differentially expressed in lung cancer cells, tissues, or liquid biopsies. Out of the 78 circRNAs conforming the panel (Table S3), 40–51 circRNAs were detected in assessed cell lines, whereas 41 and 44 were detected in early‐ and late‐stage NSCLC tissues, respectively. From the resulting circRNAs that could not be detected by nCounter, 19 could not be found in any control tissue nor in any assessed cell line (Fig. S10). Additional experiments using liquid biopsies would be of interest to address if cited circRNAs are present in such material according to our nCounter protocol, or if on the contrary, observed discrepancies may be due to the technical differences (including normalization) among the diverse platforms used in previous studies such as RNA‐seq, microarrays, or RT‐qPCR when compared to our nCounter workflow.

The resulting 13 circRNAs were detected in the cell lines with three of them, circPIK3R1, circADAM22, circCHN1 being only present in the normal epithelial cell lines (AALE and HBEC30KT). These three were described in literature to be downregulated in NSCLC [26, 27, 28, 29, 30]. From the detected circRNAs, 17 were found upregulated, according to literature review [26, 28, 29, 30], contrasting to the results achieved with nCounter; however, only three of those could be further validated by RT‐qPCR [26, 28, 30]. Although we believe that direct comparison with another circRNA panel for lung cancer detection is instrumental to fully assess the clinical utility of our panel, this was not performed due to the absence of the latter; however, this comparison will be warranted at the time other panels become available.

Most genetic analyses performed in the clinic come from paraffined specimens with either very little material or compromised quality. CircRNAs are very stable, even in this type of samples, due to their circular configuration [29]. Also, the nCounter technology performs quite well with highly degraded samples compared to other techniques since it only requires a short fragment of RNA (100 nt) for the capture and reporter probes to hybridize and emit a signal [16]. In consequence, we tested and compared results of circRNA expression from FFPE and fresh PC9 cells after nCounter analysis even if the RNA did not pass the quality control, as observed in the case of the FFPE PC9 samples, and we did obtain comparable results.

RNase‐R treatment can efficiently degrade highly structured RNA in 3′ end‐dependent manner [31]. Since most circRNAs are resistant to this exoribonuclease activity, we tested the specificity of our panel by treating cell line‐derived RNA with this enzyme prior nCounter processing. Consequently, most expressed circRNAs were enriched up to 56‐fold when compared to controls, and only five circRNAs were found affected by this treatment. Sensitivity of specific circRNAs toward the endonuclease activity of the RNase‐R enzyme was expected since it could be found reported in other publications [32]. Full or partial degradation of all linear transcripts included in the panel was observed, hence, validating the circRNA nCounter panel. 18 new circRNAs could be seen after treatment, while they could not be detected in mock‐treated samples. The degradation of the canonical mRNA which can represent up to 95% of the total RNA expression [33, 34] seems to facilitate the interaction between the circRNAs and the nCounter probes, which otherwise would be hampered by this mRNA‐induced noise, making those low‐expressed circRNA undetectable [35]. This, along with the enrichment of circRNA molecules upon linear RNA depletion suggests that this type of treatment may be particularly beneficial for the screening of circRNA (especially those with very low expression) derived from fresh material. Conversely, circRNA enrichment was not observed in treated FFPE‐derived RNA samples. As the rest of nucleic acid present in this type of material, circRNAs are crosslinked to the paraffin matrix. During the process of purification, these molecules are subject to both mechanical and chemical breakage; thus, any break in the circRNA would allow for RNAse‐R‐based degradation. As a result, we determined that this procedure can be recommended to improve circRNA detection in fresh but not paraffined specimens. However, it is imperative to mention that although RNase‐R treatment is highly recommended for circRNA screening purposes, it should be avoided in circRNA expression studies since the variability of RNAase‐R digestion efficiency for different samples may lead to biased circRNA expression quantification [36]. Therefore, untreated total RNA samples were used for the expression experiments in our study.

Since circRNA represents only 5–10% of total RNA [33, 34], different concentrations of total RNA were tested. As a result, 250 ng of total RNA proved enough for expression studies. Technical saturation was not achieved at 2000 ng of total RNA suggesting that higher concentrations could be used if analysis of transcripts expressed at lower levels is intended.

Using the explained workflow and custom‐made circRNA nCounter panel, expression analysis in lung cancer cell lines was performed. Interestingly, an overall increase in the number of circRNA raw counts was found in normal epithelial versus cancer cells. This result is in agreement with a previous study, where a global reduction of circRNA expression in cancer compared to healthy specimens was found, along with a negative correlation of overall RNA abundance and proliferation [37].

In addition, a group of differentially expressed circRNAs was discovered in the assessed cancer cell lines. Interestingly, although circPIK3R1 was downregulated in agreement with formerly published results [30], both circCHST15 and circFARSA were also downregulated. CircCHST15 was recently found highly expressed in lung cancer, correlating with PD‐L1 status and promoting immune escape of lung cancer cells [38]. Similarly, circFARSA upregulation has been described in tumor cells, promoting migration and invasion [39]. Although none of the groups used AALE nor the HBEC30KT epithelial cell line for their transcriptional analyses preventing direct comparison with our study, additional experiments with other epithelial cell lines and additional transfection studies could be of interest to shed light on the biology of these circRNAs.

Furthermore, a circRNA from the FUT8 gene which was found upregulated in cancer cells and further validated in FFPE lung cancer tissues, even at the early stage of the disease. In addition, circCHD9 and circC1orf116 were found highly expressed, while circEPB41L2, circBNC2, and circSOX13 were strongly downregulated in such material. These last three circRNAs could be further seen downregulated in NSCLC samples by RT‐qPCR validating previous nCounter results.

Circinteractome was used to further elucidate possible miRNA targets of aforementioned circRNAs.

Out of 28 predicted miRNAs for circFUT8, hsa‐mir‐186, and hsa‐miR‐1305 were the only ones presenting more than one potential binding site. Hsa‐miR‐186 was described downregulated in NSCLC, acting as an inhibitor of cancer proliferation, progression, and metastasis [40, 41], whereas hsa‐miR‐1305 was not described in any type of cancer thus far. Another mechanism of action of circFUT8 in NSCLC has been described by Zhu et al. [42] in a recent publication, where this circRNA was shown to increase proliferation, invasion, and migration of NSCLC cells via miR‐944/YESI axis.

For circCHD9, only one miRNA, hsa‐miR‐1229, was predicted. This miRNA was found upregulated in breast cancer activating β‐Catenin/Wnt signaling [43]; however, nothing has been reported to lung cancer yet. No information regarding a possible connection between circC1orf116 and this malignancy was found either. Nonetheless, this circRNA has been described to promote cell proliferation, migration, and invasion in cervical cancer by binding to miR‐518d‐5p and miR‐519‐5p and further modulating BBX8 expression [44].

Interestingly, among the several predicted miRNAs for circEPB41L2, circBNC2, and circSOX13, hsa‐miR‐942 was a common target of cited circRNAs with 4, 2, and 1 binding sites, respectively (Fig. S9). This miRNA was previously described to be involved in colorectal and esophageal cancer progression activating the Wnt/β‐catenin signaling pathway [45, 46]. However, no evidence of its role in lung cancer has been found and would require further investigation.

Lastly, further machine learning analysis of generated data using RF, GBM, and KNN algorithms provided not only a signature able to correctly classify lung cancer samples from the control specimens, with an AUC of 0.985 (RF), 0.955 (GBM), and 0.993 (KNN) using an 8‐circRNA signature, but also a 4‐circRNA signature for early‐stage lung cancer classification with comparable accuracy. These ML‐based signatures included circEPB41L2, circSOX13, circBNC2, adding evidence of the potential of mentioned circRNAs as early‐stage lung cancer biomarkers.

Since we did not perform microdissection of the tumor samples nor single cell analysis, we could not verify whether presented signature‐based circRNAs came from cancer cells or tumor microenvironment. Although this was out of our research scope since we mainly focused on the diagnostic potential of such signatures, we believe it could be of interest for future investigations. Also, most samples included in this study were lung adenocarcinomas, except for one squamous carcinoma and nine NSCLC samples with unknown histological subtype. Inclusion of different histologies in forthcoming validation studies are recommended to assess the specificity of the presented signatures. Finally, the work presented here was a proof‐of‐concept study and the main purpose was to demonstrate the feasibility of using nCounter for the study of circRNAs in lung cancer specimens. In consequence, the number of samples included was small and the abovementioned signature should be validated in a larger cohort.

## 5 Conclusions

In summary, we have developed a circRNA nCounter panel and workflow that can be used for multiplex detection of circRNA in FFPE lung cancer specimens. A cluster of differentially expressed circRNAs have been presented and further investigation is warranted to explore their potential as therapeutic targets. In addition, a 4 circRNA signature has been found through ML proving effective for early‐stage lung cancer differentiation.

These findings pave the way to future biomarker investigations and validation of liquid biopsy signatures for lung cancer detection.

## Conflict of interest

Chung‐Ying Huang and Sarah Warren were full‐time employees of NanoString Inc. at the time the study was performed. The rest of the authors declare no conflict of interests.

### Peer Review

The peer review history for this article is available at https://publons.com/publon/10.1002/1878‐0261.13182.

## Author contributions

CPV, JWPB, RR, and MAMV conceptualized and designed the experiments. MI and AAH were responsible of patient recruitment and sample collection. CPV, AGC, EA, and SRM performed the experiments. SPG, NP, CPV, performed data analysis with contributions of AFH and MH. CPV, RR and MAMV wrote the main manuscript and prepared the figures. CYH and SW contributed to reagents and materials. AFH, MH TB, MF, and CAE provided editing, comments, and experimental guidance. All authors reviewed the manuscript.

## Supporting information


**Fig. S1.** nCouter probe design allows specific recogition of the circRNAs included in the panel.

**Fig. S2.** nCounter workflow for circRNA expression studies in FFPE lun tissues.

**Fig. S3.** Reproducibility experiment comparing the log2 of normalized counts by nCounter from three independent RNA samples derived from the PC9 cell line.

**Fig. S4.** Bioanalyzer profiles of faired fresh (left) and FFPE (right) PC9 cell line‐derived RNA.

**Fig. S5.** nCounter analysis of FFPE PC9 cell line.

**Fig. S6.** Total RNA concentration assessment for circRNA analysis using the nCounter platform.

**Fig. S7.** Venn diagram showing circRNAs identified in all healthy cells (19) versus those only expressed in all lung cancer cell lines (1).

**Fig. S8.** Overall total number of raw counts in lung cells.

**Fig. S9.** Different miRNA binding sites of dysregulated circRNAs in early‐stage lung cancer tissues.

**Fig. S10.** Diagram showing the tracking of those circRNAs of the circRNA nCounter panel not detected in assessed FFPE tissues.

**Table S1.** Diagnosis and associated pathologies of the control cohort.

**Table S2.** Characteristics of FFPE samples included in the study. Tumor and lymphocyte infiltration is indicated.

**Table S3.** circRNA and mRNA candidates included in the nCounter panel.

## Data Availability

The data that support the findings of this study are available from the corresponding author (carlospedraz@icloud.com) upon reasonable request.
