# Supplementary figures and images for: Digital multiplexed analysis of circular RNAs in FFPE and fresh non‐small cell lung cancer specimens
Source: Mol Oncol. 2022 Feb 10;16(12):2367–83. doi: 10.1002/1878-0261.13182 (PMC9208080; doi:10.1002/1878-0261.13182)

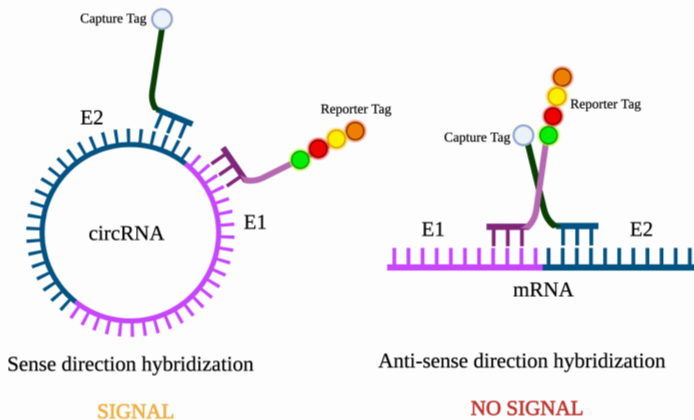

**Fig S1.** nCounter probe design allows specific recognition of the circRNAs included in the panel.

Supplement: Supplementary file 1 — Fig. S1. nCouter probe design allows specific recogition of the circRNAs included in the panel. [file MOL2-16-2367-s008.pdf]

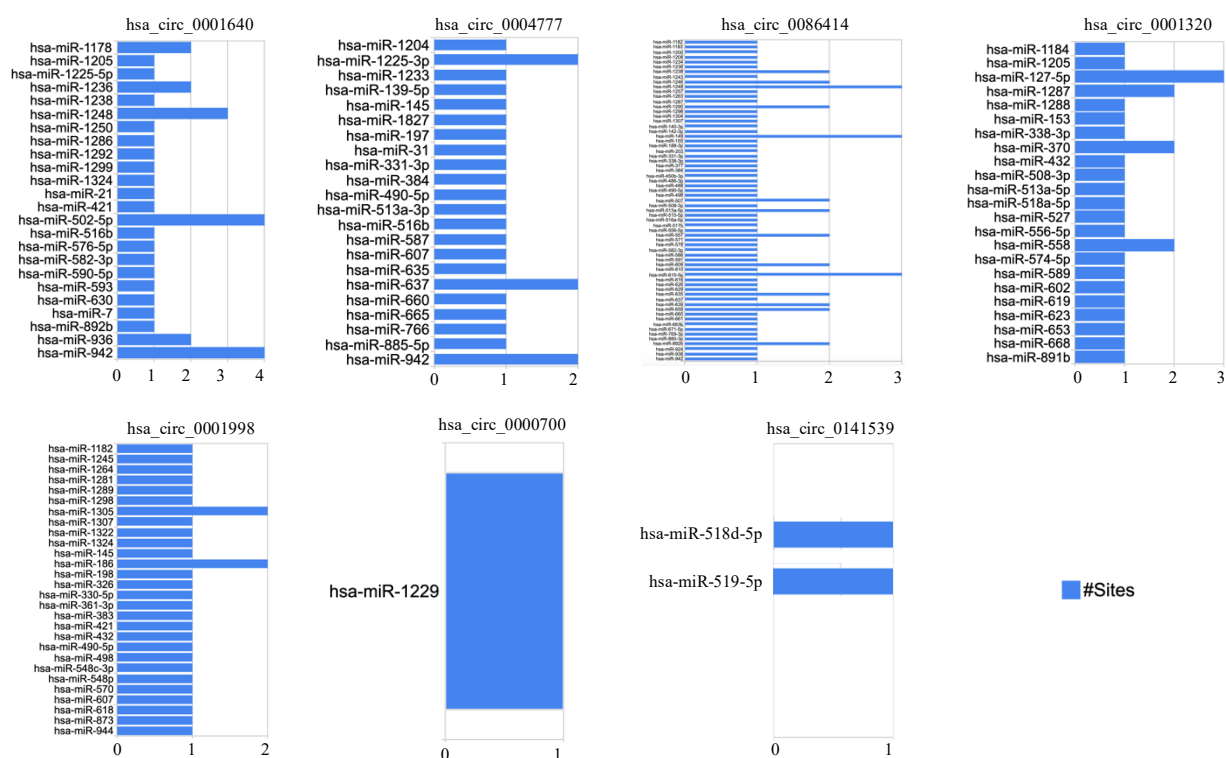

Supplement: Supplementary file 9 — Fig. S9. Different miRNA binding sites of dysregulated circRNAs in early‐stage lung cancer tissues. [file MOL2-16-2367-s004.pdf]
