# Supplementary material for: Digital multiplexed analysis of circular RNAs in FFPE and fresh non‐small cell lung cancer specimens
Source: Mol Oncol. 2022 Feb 10;16(12):2367–83. doi: 10.1002/1878-0261.13182 (PMC9208080; doi:10.1002/1878-0261.13182)
Supplement: Supplementary file 3 — Fig. S3. Reproducibility experiment comparing the log2 of normalized counts by nCounter from three independent RNA samples derived from the PC9 cell line. [file MOL2-16-2367-s007.pdf]

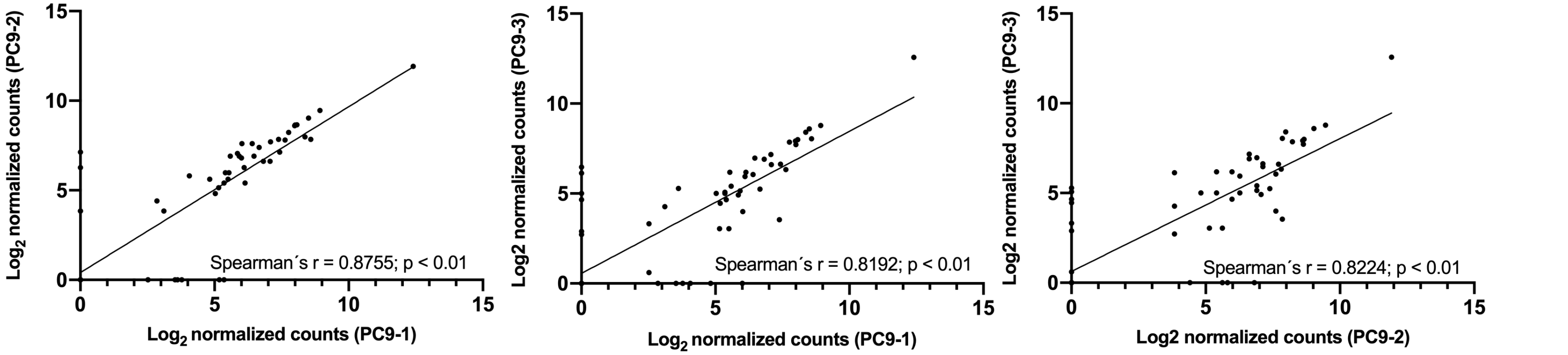

Fig S3. Reproducibility experiment comparing the log2 of normalized counts by nCounter from three independent RNA samples derived from the PC9 cell line. Spearman´s correlation coefficient is indicated.
