# Supplementary material for: Digital multiplexed analysis of circular RNAs in FFPE and fresh non‐small cell lung cancer specimens
Source: Mol Oncol. 2022 Feb 10;16(12):2367–83. doi: 10.1002/1878-0261.13182 (PMC9208080; doi:10.1002/1878-0261.13182)
Supplement: Supplementary file 4 — Fig. S4. Bioanalyzer profiles of faired fresh (left) and FFPE (right) PC9 cell line‐derived RNA. [file MOL2-16-2367-s010.pdf]

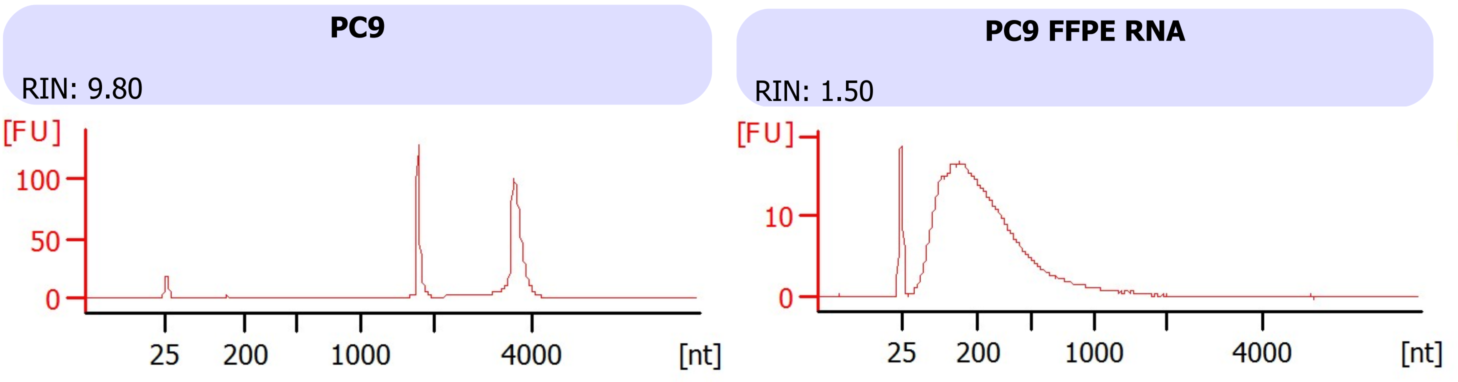

Fig S4. Bioanalyzer profiles of paired fresh (left) and FFPE (right) PC9 cell line-derived RNA. RNA integrity number (RIN) score is indicated on the upper left corner of each figure.
