# Supplementary material for: Digital multiplexed analysis of circular RNAs in FFPE and fresh non‐small cell lung cancer specimens
Source: Mol Oncol. 2022 Feb 10;16(12):2367–83. doi: 10.1002/1878-0261.13182 (PMC9208080; doi:10.1002/1878-0261.13182)
Supplement: Supplementary file 5 — Fig. S5. nCounter analysis of FFPE PC9 cell line. [file MOL2-16-2367-s006.pdf]

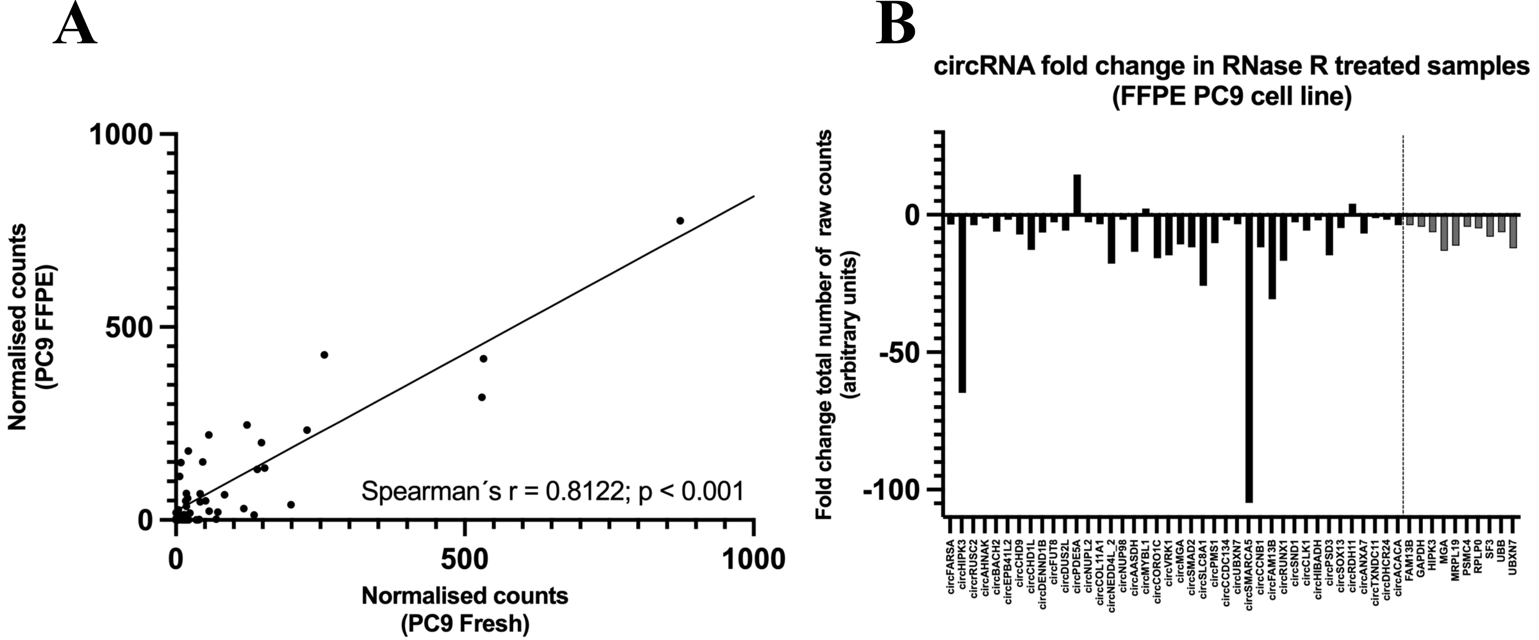

Fig S5. nCounter analysis of FFPE PC9 cell line. **A.** Correlation between circRNA from FFPE PC9 versus fresh PC9 cell lines. Spearman´s correlation coefficient is indicated. **B.** Bar plot showing circRNA and linear HK fold change after RNase R treatment.
