# Supplementary material for: Digital multiplexed analysis of circular RNAs in FFPE and fresh non‐small cell lung cancer specimens
Source: Mol Oncol. 2022 Feb 10;16(12):2367–83. doi: 10.1002/1878-0261.13182 (PMC9208080; doi:10.1002/1878-0261.13182)
Supplement: Supplementary file 6 — Fig. S6. Total RNA concentration assessment for circRNA analysis using the nCounter platform. [file MOL2-16-2367-s011.pdf]

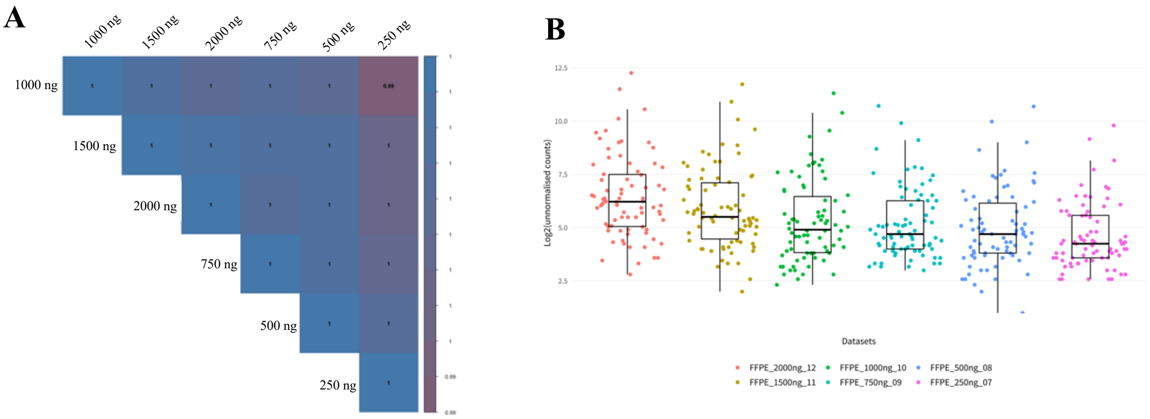

Fig S6. Total RNA concentration assessment for circRNA analysis using the nCounter platform. **A.** Figure showing the correlation of normalized counts among the different RNA concentrations tested. **B.** Boxplot of the log2 unnormalized datasets showing the count distribution in each of the different RNA concentrations tested.
