# Supplementary material for: Digital multiplexed analysis of circular RNAs in FFPE and fresh non‐small cell lung cancer specimens
Source: Mol Oncol. 2022 Feb 10;16(12):2367–83. doi: 10.1002/1878-0261.13182 (PMC9208080; doi:10.1002/1878-0261.13182)
Supplement: Supplementary file 7 — Fig. S7. Venn diagram showing circRNAs identified in all healthy cells (19) versus those only expressed in all lung cancer cell lines (1). [file MOL2-16-2367-s013.pdf]

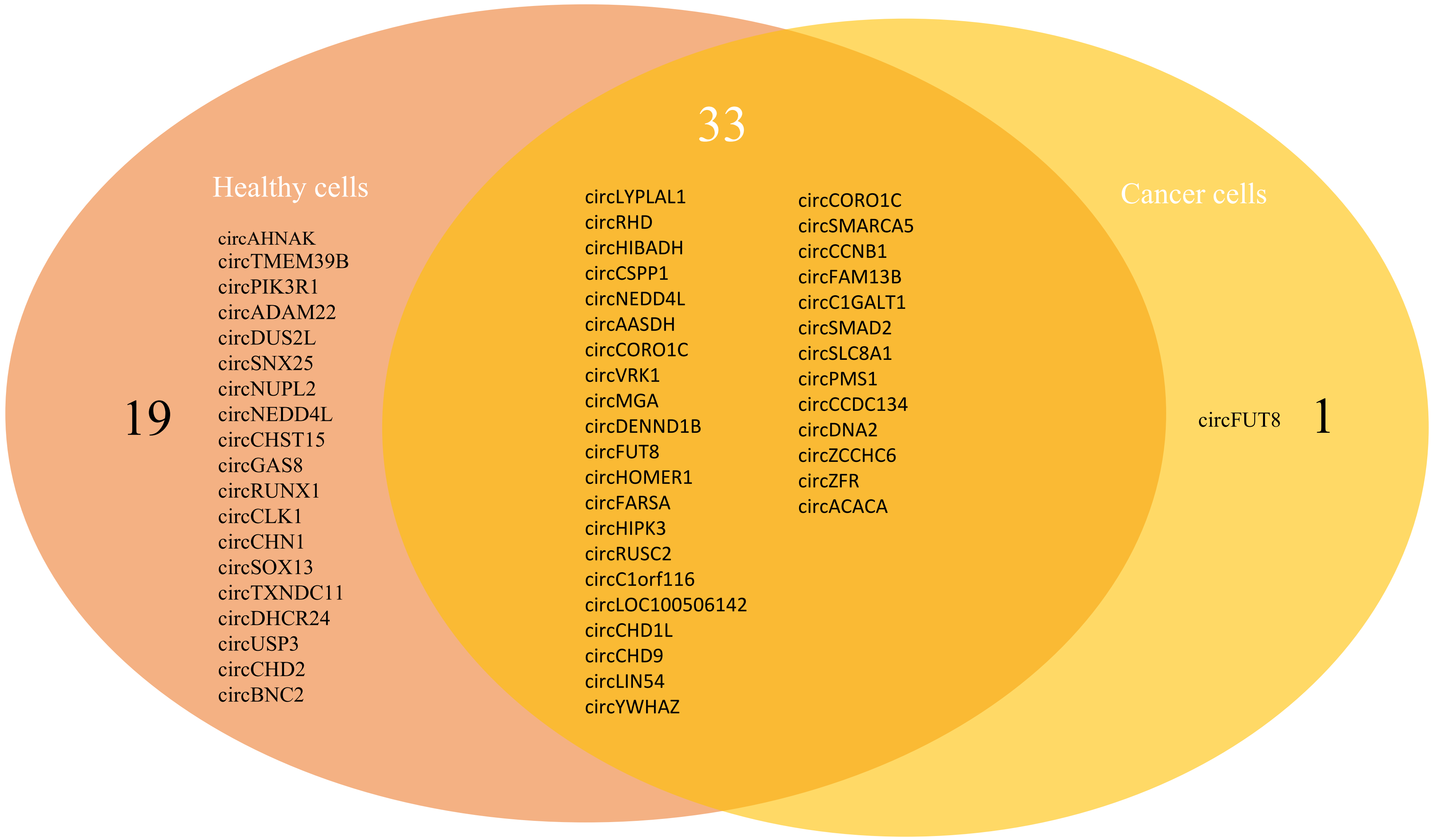

Fig. S7. Venn diagram showing circRNAs identified in all healthy cells (19) versus those only expressed in all lung cancer cell lines (1). Those circRNAs expressed in all cell lines are indicated (33).
