# Supplementary material for: Digital multiplexed analysis of circular RNAs in FFPE and fresh non‐small cell lung cancer specimens
Source: Mol Oncol. 2022 Feb 10;16(12):2367–83. doi: 10.1002/1878-0261.13182 (PMC9208080; doi:10.1002/1878-0261.13182)
Supplement: Supplementary file 8 — Fig. S8. Overall total number of raw counts in lung cells. [file MOL2-16-2367-s002.pdf]

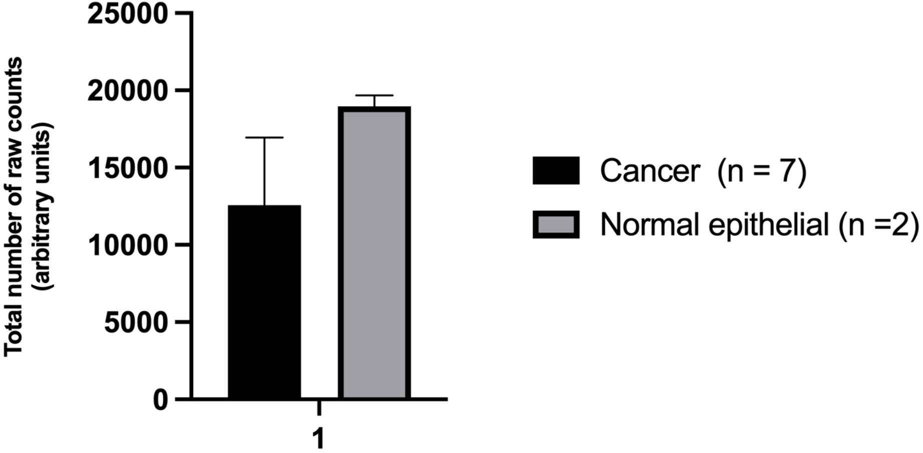

Fig S8. Overall total number of raw counts in lung cancer (A549,PC9, H2228, H3122, HOP-62, HCC-827, H1666) and normal epithelial cell lines (AALE, HBEC3KT).
