# Supplementary material for: Digital multiplexed analysis of circular RNAs in FFPE and fresh non‐small cell lung cancer specimens
Source: Mol Oncol. 2022 Feb 10;16(12):2367–83. doi: 10.1002/1878-0261.13182 (PMC9208080; doi:10.1002/1878-0261.13182)
Supplement: Supplementary file 10 — Fig. S10. Diagram showing the tracking of those circRNAs of the circRNA nCounter panel not detected in assessed FFPE tissues. [file MOL2-16-2367-s005.pdf]

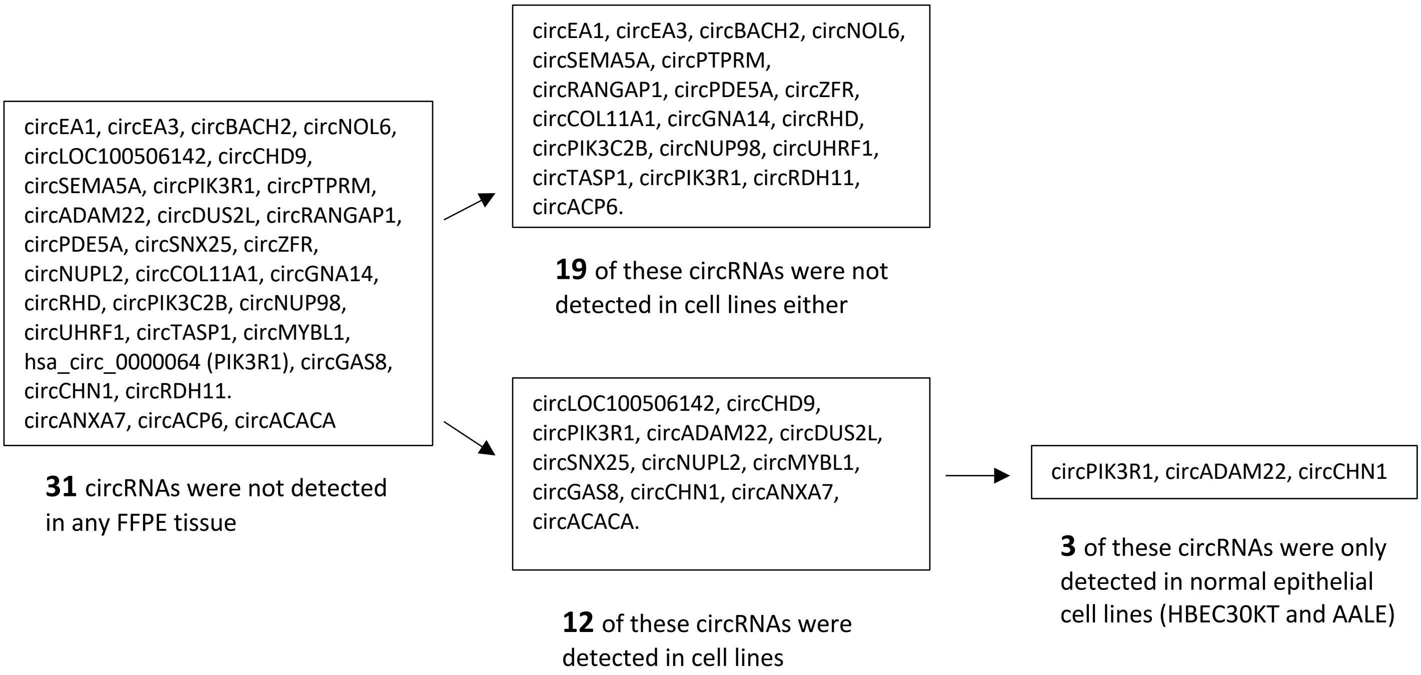

Figure S10. Diagram showing the tracking of those circRNAs of the circRNA nCounter panel not detected in assessed FFPE tissues.

Out all circRNAs included in the panel, 31 were not identified in cancer nor control FFPE tissues. 19 out of these 31 circRNAs could not be detected in any cell line. From the resting 12 that could be detected in cell lines, circPIK3R1, circADAM22 and circCHN1 could be only detected in the control cell lines (HBEC30KT and AALE).
