# Supplementary material for: Digital multiplexed analysis of circular RNAs in FFPE and fresh non‐small cell lung cancer specimens
Source: Mol Oncol. 2022 Feb 10;16(12):2367–83. doi: 10.1002/1878-0261.13182 (PMC9208080; doi:10.1002/1878-0261.13182)
Supplement: Supplementary file 11 — Table S1. Diagnosis and associated pathologies of the control cohort. [file MOL2-16-2367-s001.docx]

| **Diagnosis and associated pathologies of control cohort** | | | | | |
| --- | --- | --- | --- | --- | --- |
| **Patient ID** | **Sex** | **Age** | **Diagnosis** | **Smoking status** | **Associated pathologies** |
| IGTP-1 | M | 46 | Pulmonary congestion and emphysematous changes | Severe | Aortic insufficiency |
| IGTP-2 | M | 50 | Pneumonia | Severe | Polyglobulia, thrombopenia, moderate-severe COPD, hypothyroidism |
| IGTP-3 | M | 69 | Multiple infracentimetric, fibrous and whitish lesions in the right upper lobe, subpleural in location, suggestive of an old inflammatory process | Ex-smoker | Not information |
| IGTP-4* | F | 61 | Infiltrating acinar adenocarcinoma | Moderate | Tuberculosis in 1992 |
| IGTP-5* | F | 47 | Adenocarcinoma | Severe | Not information |
| IGTP-6* | F | 76 | Adenocarcinoma | N.I | Not information |
| IGTP-7* | M | 73 | Metastatic carcinoma | Ex-smoker | Gout disease |
| IGTP-8 | M | 29 | Bullous emphysema | N.I | Not information |
| IGTP-9 | F | 62 | Emphysema, edema and pulmonary congestion, diffuse and bilateral. | Smoker | Endoprosthesis from the arch to the proximal segment of the descending aorta with debranching of the right brachiocephalic trunk and the left common carotid artery. And the second in 2018 due to a new type A aortic dissection with severe AI, performing biological aortic replacement and substitution of the ascending aorta up to the first segment of the arch, with a Dacron tube, is complicated by endocarditis and vegetative growth in biological prosthesis, is reoperated for valve replacement and repair of the aortic ring. |
| IGTP-10 | M | 75 | No remarkable evidence. | N.I | Arteriosclerosis |
| IGTP-11 | M | 57 | Aortic rupture at the thoracic level secondary to type B aortic dissection | Severe | Obesity with a pathological history of type- B aortic dissection, in addition to severe aortic regurgitation without stenosis and Bentall-Bono surgery with ATS mechanical prosthesis and replacement of the aortic arch with implantation of a Thoraflex prosthesis. |
| IGTP-12 | M | 76 | Intestinal ischemia | N.I | Ischemic heart disease |
| IGTP-13 | M | 34 | Pulmonary embolism | N.I | Arterial hypertension, dyslipidemia, and type 2 diabetes mellitus |
| IGTP-14 | M | 47 | Dilated cardiomyopathy. | Ex-smoker | Dilated cardiomyopathy with two-vessel coronary disease, stage 3 chronic kidney disease. |
| IGTP-15 | F | N.I | N.I | N.I | Not associated pathologies |
| IGTP-16 | F | N.I | N.I | N.I | Not associated pathologies |

**Table S1.** Diagnosis and associated pathologies of the control cohort.

Different individuals with different characteristics were selected as controls to ensure that both, potential differentially expressed circRNAs and ML signatures, would be specific for lung cancer classification. N.I = Not information; F = Female; M = Male. *A non-tumoral region of the lung was used as control
