# Supplementary material for: Digital multiplexed analysis of circular RNAs in FFPE and fresh non‐small cell lung cancer specimens
Source: Mol Oncol. 2022 Feb 10;16(12):2367–83. doi: 10.1002/1878-0261.13182 (PMC9208080; doi:10.1002/1878-0261.13182)
Supplement: Supplementary file 12 — Table S2. Characteristics of FFPE samples included in the study. Tumor and lymphocyte infiltration is indicated. [file MOL2-16-2367-s003.docx]

| **Characteristics FFPE sample** | **Lung cancer patients**  **(n= 53)** | **Controls**  **(n = 16)** |
| --- | --- | --- |
| **Tumor infiltration – no. (%)** |  |  |
| 0-20 % | 1 (1.9) | - |
| 21-40 % | 6 (11.3) | - |
| 41-60 % | 13 (24.5) | - |
| 61-80 % | 16 (30.2) | - |
| 81-100 % | 4 (7.6) | - |
| Not information | 13 (24.5) | - |
| **Lymphocyte infiltration** |  |  |
| 0-5 % | 9 (17.0) | 10 (62.5) |
| 6-10 % | 14 (26.4) | 4 (25.0) |
| 11-20 % | 9 (17.0) | - |
| 21-30 % | 2 (3.8) | - |
| 31-40 % | 4 (7.5) | - |
| 40-50 % | 2 (3.8) | - |
| Not information | 13 (24.5) | 2 (12.5) |
